# An Elephant’s Gratitude to a Druggist

**Published:** 1879-09

**Authors:** 


An Elephant's Gratitude to a Druggist.—The following incident is related by an English exchange. It shows that animals are sometimes more grateful to their medical attendants than are many patients of the human species :

"Wombwell's menagerie visited Tenbury on April 28, 1874, and the elephant Lizzie, after a long walk, drank from a spring a quantity of cold water. She was much heated with the walk, and was soon afterward attacked with a violent fit of gripes, which continued for some time. Mr. John Turley, a chemist of Tenbury, who had acquired a special reputation for the treatment of animals, was sent for, and administered, with a tube, large doses of laudanum, aloes, assafœtida, turpentine and sulphuric ether. He also gave clysters frequently. The second day she was very thirsty, and drank upwards of ten gallons of linseed-tea, containing mashed potatoes and sugar. During the next two days she took nothing, and the medicine did not operate until the fourth day. Two quarts of liquid blister had also been applied to her side, and this seemed to give great relief.

"The menagerie again visited Tenbury about the middle of last month, five years having elapsed. As the procession moved down the street, the elephant recognized, at his shop door, her former doctor. With a refinement of feeling which does her infinite credit, she crossed the road and placed her trunk in Mr. Turley's hand, making a peculiar grunting noise as if she were pleased. Mr. Turley visited the exhibition, and was again greeted with every demonstration of affection, the embraces of Lizzie at first occasioning some alarm. The narrative is a very remarkable one, and is quite worthy of a place among the stories of animal intelligence which have been handed down to us from Androcles onward."—*Drug. Circular.*

